# Efficacy of Taiji imagery therapy in stroke patients: A systematic review and meta-analysis

**DOI:** 10.1097/MD.0000000000044169

**Published:** 2025-08-22

**Authors:** Beihai Ge, Jianjian Zhu, Fu Zhang, Xianyan Han, Yaqian Xia, Qiangze Ji, Peili Sun, Yuqing Li, Huiying Zhang, Jianghong Guo

**Affiliations:** a Department of Neurology, Rugao Affiliated Hospital of Nantong University, Rugao People’s Hospital, Nantong, Jiangsu, People’s Republic of China; b Department of Cardiology, Rugao Affiliated Hospital of Nantong University, Rugao People’s Hospital, Nantong, Jiangsu, People’s Republic of China.

**Keywords:** imagery therapy, meta-analysis, randomized controlled trial, stroke, systematic review, Taiji

## Abstract

**Background:**

To explore the efficacy of Taiji imagery therapy (TIT), a combination of Taiji and imagery therapy, in treating stroke patients.

**Methods:**

Suitable studies for the meta-analysis were retrieved from 8 research databases: China National Knowledge Infrastructure, SinoMed, China Wanfang, Chongqing VIP Information, Web of Science, PubMed, Cochrane Library, and EMBASE (date of retrieval: October 26, 2024). Articles were searched manually and independently by 2 researchers. Other researchers evaluated the included articles using the modified Physiotherapy Evidence Database scale. Using STATA software (version 14.0) for data analysis, the combined effect size was used to calculate forest plots, sensitivity analysis was used to explore the source of heterogeneity, and Egger test was used to detect publication bias.

**Results:**

The literature-based meta-analysis included 422 stroke patients from 7 studies. The findings revealed that TIT significantly increased upper limb motor function (mean difference [MD] = 2.88, 95% confidence interval [CI]: 2.33–3.44, *P* < .001, *I*^2^ = 0%), lower limb motor function (MD = 3.98, 95% CI: 0.45–7.52, *P* = .027, *I*^2^ = 95.1%), and activities of daily living (MD = 10.65, 95% CI: 3.82–17.49, *P* < .001, *I*^2^ = 95.0%). The sources of heterogeneity can be explained by the frequency, time, and duration of the study. No publication bias was observed in this study.

**Conclusions:**

TIT positively affected upper limb motor function, lower limb motor function, and activities of daily living in stroke patients. In future, we aim to include TIT in randomized controlled trials of the same type to increase the overall number of studies.

## 1. Introduction

As society continues to develop rapidly and with fast work patterns, people have prioritized work, salary, and position, among others, over health. According to a study, approximately 12.2 million people are affected by stroke globally.^[1]^ Stroke is an acute cerebrovascular disease, which is a group of diseases caused by sudden rupture of brain blood vessels or blockage of blood vessels, preventing blood flow into the brain, resulting in brain tissue damage. There are several important risk factors for stroke, such as atrial fibrillation, diabetes, hypertension, atherosclerosis, dissections, etc.^[2]^ Stroke can lead to loss of balance or coordination ability, numbness of limbs, loss of speech ability, and facial asymmetry.^[3]^ It mainly affects middle-aged and older populations; this population is therefore at the high risk for poor outcomes because of the defect of neurological function, influencing their quality of life.^[4]^

Taiji is a mind-body practice combining slow movements, meditation, and deep breathing.^[5]^ Imagery therapy (IT), a new and novel method developed in the 1970s in China, is used to eliminate memory images of psychological and physical symptoms to relax the mind and muscles of the body to treat conditions such as trauma, dementia, and stroke.^[6–8]^ TIT, a combination of Taiji and IT that originated from ancient China, has been used, although not widely, for treating stroke.^[5,9,10]^ However, there are limited trials regarding the efficiency of TIT in stroke, with no meta-analysis on this topic. Patients with stroke experience varying degrees of functional impairment.^[11,12]^ Specific to Taiji, we found a significant amount of evidence supporting its efficiency in stroke treatment.^[13–15]^ Similarly, we found that IT had a positive effect on the upper limbs,^[16]^ but not on the lower limbs motor function, of patients with stroke.^[16]^ We found no other evidence to prove the benefits of IT in other aspects of non-acute ischemic and hemorrhagic stroke.

Therefore, in this study, we performed a systematic review and meta-analysis of high-quality randomized controlled trials (RCTs) to determine the efficacy of TIT in patients with stroke.

## 2. Methods

*Registration*: This systematic review and meta-analysis was conducted and reported in line with the Preferred Reported Items for Systematic Reviews and Meta-Analysis statement^[17]^ and has been registered in PROSPERO (registration number: CRD42023461389).

*Inclusion criteria*: Type of study: RCTs from peer-reviewed journals; type of participants: patients with stroke, confirmed by computed tomography or magnetic resonance imaging; type of intervention: experimental group (TIT + other treatments) and control group (other treatments); type of outcome measures: health outcomes with at least 2 articles. After carefully screening the literature, we used 4 scales to measure health outcomes: Fugl–Meyer motor assessment–upper extremity for upper limb motor function (ULMF), Fugl–Meyer motor assessment-lower extremity for lower limb motor function, Berg Balance Scale for balance ability (BA), and Modified Barthel Index for activities of daily living (ADL).

*Exclusion criteria*: Severe loss or disappearance of proprioceptive and tactile sensations and cognitive impairment; presence of other complications restricting activity; research with incomplete data; scientific meetings, research abstracts, and research from a medical specialty meeting; and non-RCTs.

*Information sources and searches*: Eight research databases were used for retrieval: China National Knowledge Infrastructure, SinoMed, China Wanfang, Chongqing VIP Information, Web of Science, PubMed, Cochrane Library, and EMBASE (date of retrieval: October 26, 2024). Articles were manually searched by several researchers (B.G. and J.G.). The search terms are shown in Table 1, and the search strategy in PubMed is shown in Table S1, Supplemental Digital Content, https://links.lww.com/MD/P776. Literature retrieval was conducted by other researchers (J.Z., F.Z., X.H., Y.X., Z.J., P.S., Y.L., and H.Z.) to ensure accuracy.

**Table 1:** Search words.

MeSH words	Taiji	Free words	Tai-Ji, Tai Chi, Tai Ji Quan, Taijiquan, Tai Chi Chuan
	Stroke		Cerebrovascular accident (CVA), cerebrovascular apoplexy, apoplexy, cerebrovascular, vascular accident, brain vascular accident, cerebrovascular stroke, cerebral stroke, acute stroke, acute cerebrovascular accident
	RCT		Randomized, placebo, randomized controlled trial

CVA = cerebrovascular accident, RCT = randomized controlled trial.

*Data collection process*: Data were extracted using the predefined data extraction form by 2 researchers, followed by a cross-check. Any disagreements were discussed between any 2 researchers, and if no consensus was reached, a third researcher was involved. The extracted data included the following: general characteristics and data of the included articles, including the first author’s last name, year of publication, sample size, patient’s age, sex cohort size, details of the experiment (frequency, time, and duration), methods used in the experimental or control group, and indicators of the outcomes and average ± standard deviation of the experimental and control groups. Finally, a third researcher cross-checked the data to avoid any potential human errors.

*Risk of bias across studies*: Any 2 researchers used a modified Physiotherapy Evidence Database scale to evaluate the included articles. As previously mentioned, any disagreements were discussed by 2 researchers, and if no consensus was reached, a third researcher was involved.

*Statistical analysis*: Data were analyzed using the STATA software (version 14.0) (STATA Corp, College Station ). First, a random effects model was used. Second, heterogeneity was assessed using *I*^2^ and *Q* tests; low heterogeneity was observed when *I*^2^ was ≤50% and *P* was >.1, and high heterogeneity was observed when *I*^2^ was >50% and *P* was <.1. Third, the mean difference (MD) and 95% confidence interval (CI) were used. Finally, the combined effect size was used to calculate the forest plots, sensitivity analysis to explore the source of heterogeneity, and Egger test to detect publication bias.

## 3. Results

**Figure 1.**
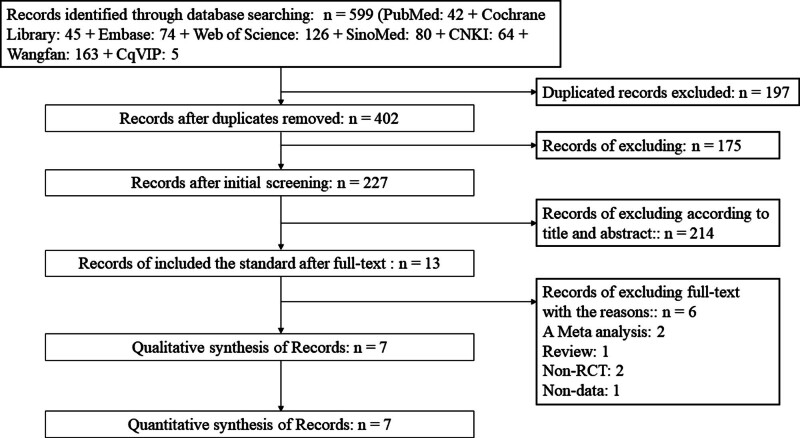
Flowchart of the study selection.

*Study selection*: A total of 599 articles were selected. After excluding 197 duplicates, 402 studies remained. Of the 402, 175 articles were removed after the initial screening. After reviewing the titles and abstracts, 214 articles were removed, leaving 13 for full-text reading. After screening based on article type and data integrity, 7 articles^[5,9,10,18–21]^ were finally considered for qualitative and quantitative synthesis (Fig. 1).

*General characteristics of the included articles*: The general characteristics of the 7 RCTs, including 422 patients with stroke, considered for the analysis are presented in Table 2. The publication dates of the included articles were 2014 to 2022. Of the 422 participants, 235 were female, 159 were male, and 28 had no data on their gender. In terms of the intervention frequency of the experimental group, all were ≥5 times per week, of which 4 articles were 7 times per week. In terms of time per intervention, all participants were between 20 and 50 minutes. In terms of the total duration of intervention, the shortest was 2 weeks, and the longest was 8 weeks. In terms of the intervention methods used, the experimental group was given TIT + normal therapy and the control group was given only normal therapy in 6 articles. The remaining study (one) used Therapeutic Bicycle Training.

**Table 2:** Basic characteristics of the included articles.

References	Sample size (E/C)	Age* (E/C, year)	Gender (F/M)	Disease duration* (E/C, month)	Experiment			Methods (E/C)	Outcomes
					Frequency	Time	Duration		
Zhang et al^[9]^	16/16	59.13 ± 13.37	E: 10/6	3.00 ± 1.68	7 times/week	30 min	3 weeks	E: Taiji imagery therapy + normal therapy	FMA-UE, MBI
		63.63 ± 6.73	C: 14/2	3.72 ± 1.87				C: normal therapy	
Zhang et al^[18]^	20/20	67.80 ± 12.22	E: 10/10	2.00 ± 1.67	7 times/week	30 min	3 weeks	E: Taiji imagery therapy + normal therapy	FMA-LE
		66.65 ± 10.49	C: 11/9	3.38 ± 1.97				C: Normal therapy	
Yuan^[10],^†	28/31	57.39 ± 6.9	E: 22/9	9.06 ± 2.41‡	5 times/week	44–50 min	8 weeks	E: Taiji imagery therapy + normal therapy	FAM-LE
		58.06 ± 6.47	C: 21/10	9.71 ± 2.12‡				C: normal therapy	
Pan^[19],^†	33/32	57.58 ± 8.75	E: 12/21	62.52 ± 5.77‡	6 times/week	20 min	6 weeks	E: Taiji imagery therapy + normal therapy	BBS
		61.63 ± 8.62	C: 10/22	63.25 ± 8.94‡				C: normal therapy	
Xu^[20]^,†	26/26	61.00 ± 10.23	E: 17/9	/	7 times/week	45 min	2 weeks	E: Taiji imagery therapy + normal therapy	FMA-UE
		63.65 ± 11.30	C: 20/6	/				C: normal therapy	
Li et al^[21]^	44/44	63.28 ± 6.82	E: 26/18	/	5 times/week	40 min	6 weeks	E: Taiji imagery therapy + normal therapy + therapeutic bicycle training	MA-LE, BBS, MBI
		62.75 ± 6.68	C: 28/16	/				C: normal therapy + normal therapy + therapeutic bicycle training	
Luo et al^[8]^	43/43	65.77 ± 5.36	E: 29/14	61.61 ± 3.72‡	7 times/week	30 min	8 weeks	E: Taiji imagery therapy + normal therapy	FMA-LE, BBS, MBI
		5:163:161:16	C: 27/16	63.25 ± 8.94‡				C: normal therapy	

BBS = Berg Balance Scale, C = control group, E = experimental group, F = female, FMA-LE = Fugl–Meyer motor assessment-lower extremity, FMA-UE = Fugl–Meyer motor assessment-upper extremity, M = male, MBI = Modified Barthel Index.

* Data are expressed as mean ± standard deviation.

† On behalf of the dissertation.

‡ On behalf of days.

*Quality assessment*: The Physiotherapy Evidence Database scale was used to assess the included articles. We removed items 5 and 6 from the original version, providing a full score of 9 points for evaluation. Seven articles included in this study scored 7 points (Table 3). The methodological quality assessment of these articles indicated a high-quality rating for the included articles; consequently, the quality of the results was also high.

**Table 3:** Quality assessment of the included articles.

References	Item 1	Item 2	Item 3	Item 4	Item 5	Item 6	Item 7	Item8	Item 9	Total score
Zhang et al^[9]^	1	1	0	1	0	1	1	1	1	7
Zhang et al^[18]^	1	1	0	1	0	1	1	1	1	7
Yuan^[10]^	1	1	0	1	0	1	1	1	1	7
Pan^[19]^	1	1	0	1	0	1	1	1	1	7
Xu^[20]^	1	1	0	1	0	1	1	1	1	7
Li et al^[21]^	1	1	0	1	0	1	1	1	1	7
Luo et al^[8]^	1	1	0	1	0	1	1	1	1	7

### 3.1. Primary outcomes

**Figure 2.**
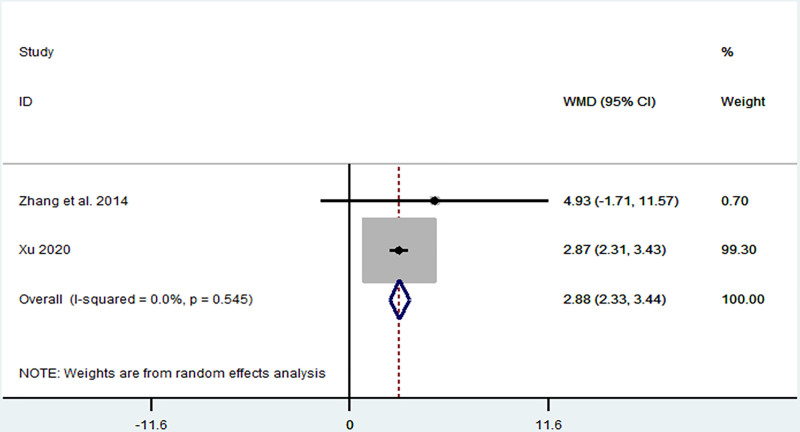
Forest plot representing the association between Taiji imagery therapy and upper limb motor function.

*ULMF:* ULMF was reported in 2 articles,^[18,20]^ which included 84 patients. The heterogeneity was low (*I*^2^ = 0%, *P* = .37), and we found that TIT significantly increased ULMF (MD = 2.88, 95% CI: 2.33–3.44, *P* < .001) (Fig. 2).

**Figure 3.**
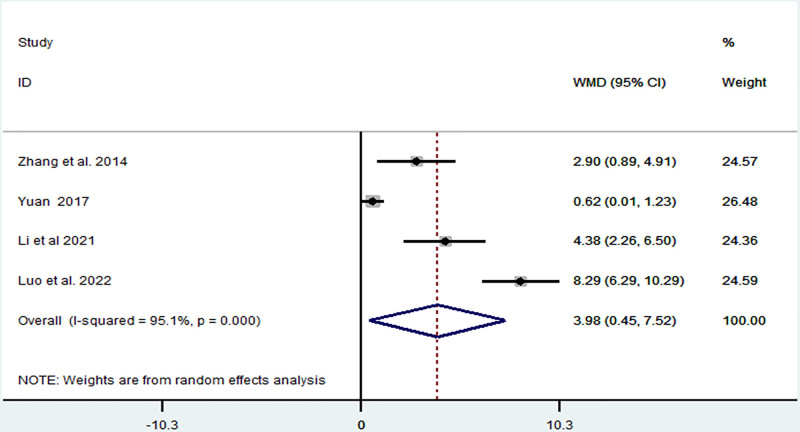
Forest plot representing the association between Taiji imagery therapy and lower limb motor function.

**Figure 4.**
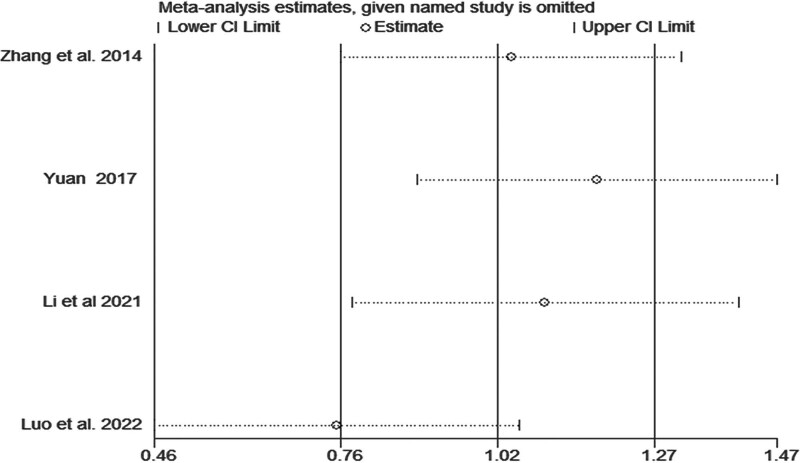
Sensitivity analysis of the association between Taiji imagery therapy and lower limb motor function.

*LLMF:* LLMF was reported in 4 articles,^[5,9,10,21]^ which included 273 patients. The heterogeneity was high (*I*^2^ = 95.1%, *P* < .1), and we found that TIT increased LLMF (MD = 3.98, 95% CI: 0.45–7.52, *P* = .027) (Fig. 3). We explored the source of heterogeneity using sensitivity analysis. We found that the article by Luo et al^[8]^ was different from other articles, as shown in Fig. 4. After removing this article,^[5]^ the heterogeneity decreased from 95.1% to 86.4%, and TIT increased LLMF (MD = 2.47, 95% CI: 0.03–4.92, *P* = .047). Therefore, the frequency, time, and duration of the experimental group could be sources of heterogeneity.

**Figure 5.**
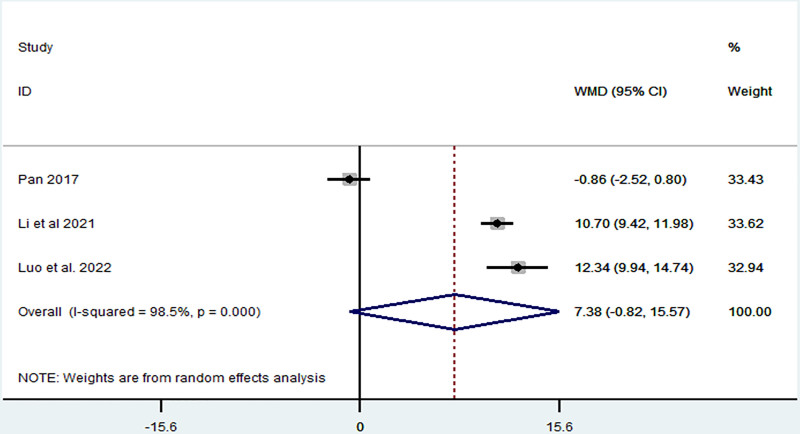
Forest plot representing the association between Taiji imagery therapy and balance ability.

**Figure 6.**
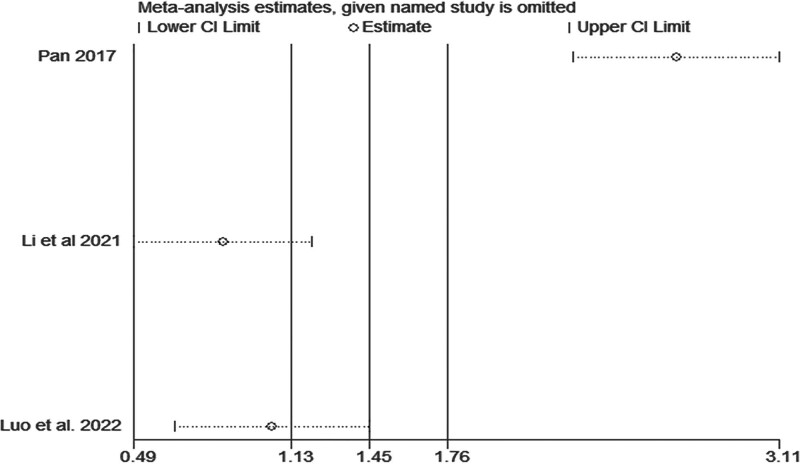
Sensitivity analysis of the association between Taiji imagery therapy and balance ability.

*BA:* BA was reported in 3 articles,^[5,19,21]^ which included 240 patients. Heterogeneity was high (*I*^2^ = 98.5%, *P* < .1); however, TIT did not influence BA (MD = 7.38, 95% CI: −0.82–15.57, *P* = .078) (Fig. 5). We found that the article by Pan was different from other articles, as shown in Fig. 6. After removing this article,^[19]^ the heterogeneity decreased from 98.5% to 28.5%, and TIT significantly increased the BA (MD = 11.19, 95% CI: 9.72–12.67, *P* < .001). This indicated that the time of the experimental group could be the source of heterogeneity.

**Figure 7.**
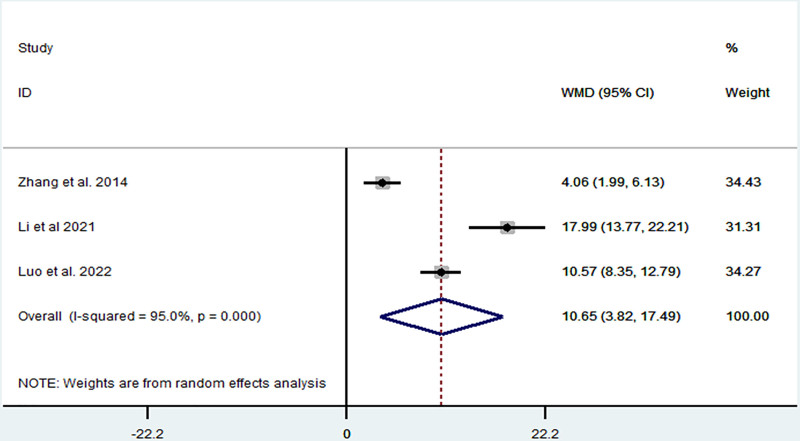
Forest plot representing the association between Taiji imagery therapy and activity of daily living.

**Figure 8.**
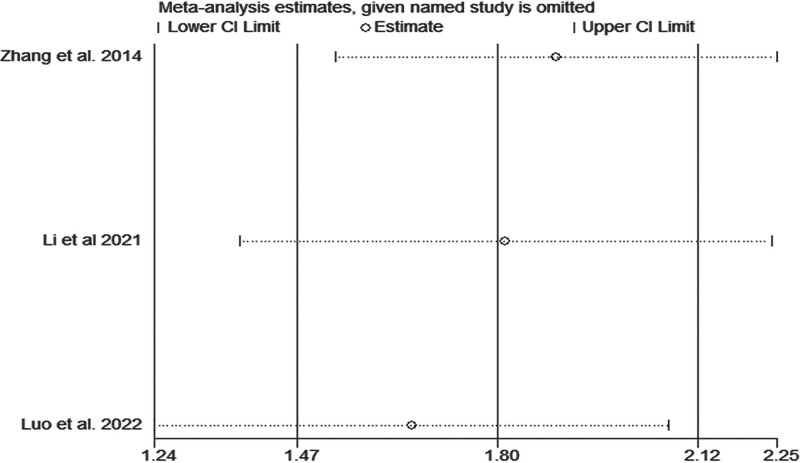
Sensitivity analysis of the association between Taiji imagery therapy and activity of daily living.

*ADL:* ADL was reported in 3 articles,^[5,18,21]^ which included 206 patients. Heterogeneity was high (*I*^2^ = 95.0%, *P* < .1), and TIT significantly increased ADL (MD = 10.65, 95% CI: 3.82–17.49, *P* < .001) (Fig. 7). We found that these 3 articles^[5,18,21]^ were not significantly different from the other articles, as shown in Fig. 8.

**Figure 9.**
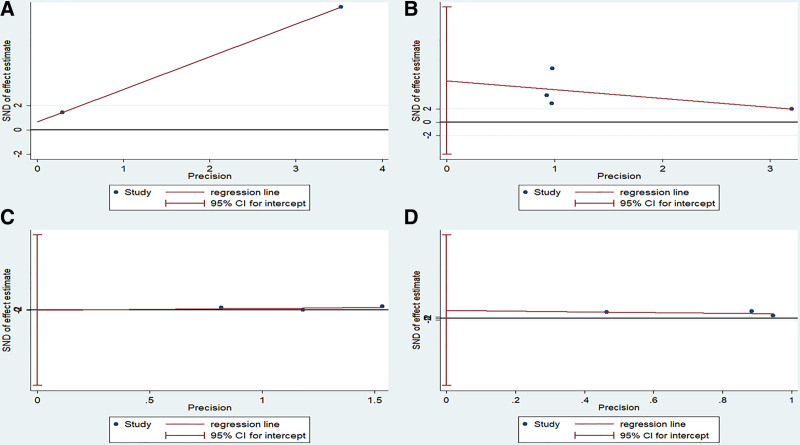
Egger test of the association between Taiji imagery therapy and upper limb motor function (A), lower limb motor function (B), balance ability (C), and activity of daily living (D).

*Publication bias of the included articles*: The Egger test indicated that this study had no publication bias (Fig. 9A–D). Because ULMF was reported in only 2 articles, the *P*-value could not be obtained. In terms of LLMF, BA, and ADL, the *P* values were .14, .96, and .40, respectively.

## 4. Discussion

In this study, we systematically reviewed 7 RCTs. Forest plots, sensitivity analysis, and publication bias were used to test the efficiency of TIT in stroke patients. We found that TIT had a significantly positive effect on ULMF (MD = 2.88, *P* < .001), ADL (MD = 10.65, *P* < .001), and LLMF (MD = 3.98, *P* = .027). However, for BA (MD = 7.38, *P* = .078), the results were not stable after sensitivity analysis (MD = 11.19, *P* < .001). This study aimed to explore the efficiency of TIT in stroke patients, which the findings of the meta-analysis did not support. TIT is a relatively new treatment for stroke. For type 2 diabetes and the older population, we found limited evidence to prove its efficacy.^[22,23]^

ULMF is an important health indicator in stroke patients. We found that TIT significantly improved the ULMF in patients with stroke, which is consistent with the findings of a previous study,^[24]^ in which the experimental group used IT. Currently, there are limited studies investigating the effects of TIT in patients with stroke; therefore, the risk of publication bias should also be considered in the future. In clinical practice, TIT is a new, low-cost, and effective treatment for stroke patients. Studies have shown that Taiji has a positive effect on motor function, mental health, and physical health in patients with stroke.^[25–27]^ Some advantages of Taiji include low cost, ease of use, and ease of accessibility.^[28]^

LLMF is as important as ULMF in patients with stroke. We found that TIT also improved LLMF in these patients. Furthermore, we found that mirror therapy had a positive effect on LLMF in this population.^[29,30]^ However, mirror therapy is not a form of TIT. Nevertheless, further studies investigating the effects of TIT on LLMF are warranted to test the stability of this result.

Previous studies have demonstrated a positive effect of Taiji on BA^[31]^ but not TIT. It is important to note that Taiji and TIT are not the same: Taiji is a form of physical exercise, whereas TIT relies on mental exercise for movement. This may be the main reason why TIT did not positively affect the BA.

TIT had a significantly positive effect on ADL in patients with stroke. These patients tend to have depressed mood.^[13]^ Although we found no studies using Taiji or TIT for improving ADL, ADL in patients with stroke is an important aspect that should always be considered. Further studies are required to test the stability of these results.

One of the strengths of this study was that it focused on TIT, a relatively new treatment for stroke, compared to Taiji. Second, this is the first systematic review and meta-analysis to show TIT as a positive method for improving ULMF, LLMF, and ADL in patients with stroke. Third, the included studies were of high quality, indicating that the level of evidence in this study was high. Finally, the study had no publication bias was observed. However, this study had some limitations. First, it included only 7 articles, making the sample size relatively small. Second, those patients with cognitive impairment and severe sensory deficits were not excluded in our study. Third, the experimental group was combined with other treatments, which may have had a synergistic effect.

## 5. Conclusions

This systematic review and meta-analysis showed that TIT positively affected ULMF, LLMF, and ADL in patients with stroke. In the future, we will consider designing a TIT with RCTs of the same type to increase the overall number of studies.

## Author contributions

**Conceptualization:** Beihai Ge, Jianghong Guo.

**Data curation:** Jianghong Guo.

**Formal analysis:** Jianghong Guo.

**Funding acquisition:** Jianghong Guo.

**Methodology:** Peili Sun, Huiying Zhang.

**Project administration:** Jianjian Zhu, Fu Zhang, Xianyan Han, Peili Sun.

**Resources:** Jianjian Zhu, Xianyan Han, Qiangze Ji.

**Software:** Jianjian Zhu, Fu Zhang.

**Supervision:** Fu Zhang, Xianyan Han, Qiangze Ji, Yuqing Li, Huiying Zhang.

**Validation:** Fu Zhang, Xianyan Han, Yaqian Xia, Huiying Zhang.

**Visualization:** Beihai Ge, Yaqian Xia.

**Writing – original draft:** Beihai Ge.

**Writing – review & editing:** Yaqian Xia, Qiangze Ji, Peili Sun, Yuqing Li.

## Supplementary Material


