# Supplementary material for: Efficacy of Taiji imagery therapy in stroke patients: A systematic review and meta-analysis
Source: Medicine (Baltimore). 2025 Aug 22;104(34):e44169. doi: 10.1097/MD.0000000000044169 (PMC12385041; doi:10.1097/MD.0000000000044169)
Supplement: Supplementary file 1 [file medi-104-e44169-s001.docx]

PubMed

#10 #7 AND #8 AND #9

#9 #3 OR #6

#8 #2 OR #5

#7 #1 OR #4

#6 Randomized Controlled Trial[Title/Abstract] OR Randomized[Title/Abstract] OR Placebo[Title/Abstract] OR RCT[Title/Abstract]

#5 Strokes[Title/Abstract] OR Cerebrovascular Accident[Title/Abstract] OR Cerebrovascular Accidents[Title/Abstract] OR CVA (Cerebrovascular Accident[Title/Abstract]) OR CVAs (Cerebrovascular Accident[Title/Abstract]) OR Cerebrovascular Apoplexy[Title/Abstract] OR Apoplexy, Cerebrovascular[Title/Abstract] OR Vascular Accident, Brain[Title/Abstract] OR Brain Vascular Accident[Title/Abstract] OR Brain Vascular Accidents[Title/Abstract] OR Vascular Accidents, Brain[Title/Abstract] OR Cerebrovascular Stroke[Title/Abstract] OR Cerebrovascular Strokes[Title/Abstract] OR Stroke, Cerebrovascular[Title/Abstract] OR Strokes, Cerebrovascular[Title/Abstract] OR Apoplexy[Title/Abstract] OR Cerebral Stroke[Title/Abstract] OR Cerebral Strokes[Title/Abstract] OR Stroke, Cerebral[Title/Abstract] OR Strokes, Cerebral[Title/Abstract] OR Stroke, Acute[Title/Abstract] OR Acute Stroke[Title/Abstract] OR Acute Strokes[Title/Abstract] OR Strokes, Acute[Title/Abstract] OR Cerebrovascular Accident, Acute[Title/Abstract] OR Acute Cerebrovascular Accident[Title/Abstract] OR Acute Cerebrovascular Accidents[Title/Abstract] OR Cerebrovascular Accidents, Acute[Title/Abstract]

#4 Tai-ji[Title/Abstract] OR Tai Chi[Title/Abstract] OR Chi, Tai[Title/Abstract] OR Tai Ji Quan[Title/Abstract] OR Ji Quan, Tai[Title/Abstract] OR Quan, Tai Ji[Title/Abstract] OR Taiji[Title/Abstract] OR Taijiquan[Title/Abstract] OR T'ai Chi[Title/Abstract] OR Tai Chi Chuan[Title/Abstract]

#3 "Randomized Controlled Trial" [Publication Type]

#2 "Stroke"[Mesh]

#1 "Tai Ji"[Mesh]
